# Comparative Study of Temporal Changes in Pigments and Optical Properties in Sepals of *Helleborus odorus* and *H. niger* from Prebloom to Seed Production

**DOI:** 10.3390/plants11010119

**Published:** 2021-12-31

**Authors:** Mateja Grašič, Maja Dacar, Alenka Gaberščik

**Affiliations:** Department of Biology, Biotechnical Faculty, University of Ljubljana, SI-1000 Ljubljana, Slovenia; c.maja.dacar@gmail.com (M.D.); alenka.gaberscik@bf.uni-lj.si (A.G.)

**Keywords:** understorey, *Helleborus niger*, *Helleborus odorus*, spring geophyte, sepals, optical properties, photochemical efficiency

## Abstract

*Helleborus niger* is an evergreen species, while *H. odorus* is an herbaceous understorey species. They both develop flowers before the forest canopy layer closes. Their sepals remain after flowering and have multiple biological functions. To further elucidate the functions of sepals during flower development, we examined their optical and chemical properties, and the photochemical efficiency of photosystem II in the developing, flowering, and fruiting flowers. Sepals of the two species differed significantly in the contents of photosynthetic pigments and anthocyanins, but less in the UV-absorbing substances’ contents. Significant differences in photosynthetic pigment contents were also revealed within different developmental phases. The sepal potential photochemical efficiency of photosystem II was high in all developmental phases in *H. odorus*, whereas in *H. niger*, it was initially low and later increased. In the green *H. odorus* sepals, we obtained typical green leaf spectra with peaks in the green and NIR regions, and a low reflectance and transmittance in the UV region. On the other hand, in the white *H. niger* sepals in the developing and flowering phases, the response was relatively constant along the visible and NIR regions. Pigment profiles, especially chlorophylls, were shown to be important in shaping sepal optical properties, which confirms their role in light harvesting. All significant parameters together accounted for 44% and 34% of the reflectance and transmittance spectra variability, respectively. These results may contribute to the selection of *Helleborus* species and to a greater understanding of the ecological diversity of understorey plants in the forests.

## 1. Introduction

In temperate deciduous forests, pronounced changes in light conditions during the growing season occur due to leaf development and tree canopy closing [1]. In early spring, the forest floor is initially exposed to full sunlight, and is then subject to significantly reduced solar radiation later in the growing season, leading to changes in understorey plant species [2]. These plant species differ significantly in their life cycles and can be generally divided into three groups: (1) species that develop all organs in early spring before canopy closure, (2) species that develop leaves prior to canopy closure, while flowers and fruits develop after canopy closure, and (3) those that flower and fruit after canopy closure, as is the case for late summer species [3]. The representatives of the first plant group possess a variety of traits that support quick development in the period with abundant light [4]. The development of flowers before leaf unfolding depends on carbohydrates accumulated in the underground organs [1]. In addition, some of the plant species have evergreen leaves [5], while others have green organs other than leaves, such as bracts and sepals [6]. These green structures contribute significantly to the plant energy budget [7,8], since they harvest solar energy in the early season, before canopy leaves unfold [6]. Understorey plant species also respond to changing light conditions [9] by developing traits that adjust their light-use efficiency [2,10]. These traits determine the optical properties of their structures, namely the ratio of the light reflected, absorbed, and transmitted through a specific plant organ [2,11,12]. Optical properties can be either tissue- or species-specific, or they can vary according to tissue ontogenetic development and species phenotypic plasticity related to changes in the environmental conditions in the habitat [12,13].

Among the plants that use the advantage of high light conditions in late winter and early spring are also different representatives of the genus *Helleborus*, which consists of 22 species of herbaceous or evergreen perennial plants that originate from Europe and Asia, with Balkan Peninsula being a diversity hotspot of the genus [14,15]. Flowers of the *Helleborus* species do not consist of petals, but of sepals, while petals are transformed into nectaries. They may substitute the multiple roles of petals, as they attract pollinators, protect flowers, and regulate flower temperature [16]. *Helleborus* flowers are epiphyllous, non-specialised, and thus, are pollinated by a variety of insects, such as honeybees, bumblebees, different flies, and even wasps, ants, beetles, representatives of the genus *Thysanoptera*, and possibly wind, as in the case of *H. niger* [17]. A study of pollinator visits in *H. foetidus* and *H. bocconei* showed that their pollinators were mainly representatives of the genus *Bombus* [18]. In some species, flower colour changes during the development and senescence. In *H. orientalis* cv. *Olympicus*, it was shown that creamy white sepals turned green at later stages [19]. It was also shown that sepals of pollinated flowers were richer in chlorophyll in comparison to non-pollinated flowers and senescent sepals [20].

In this study, we aimed to examine the optical and chemical properties of sepals of two *Helleborus* species that are native to Slovenia, namely *H. niger* L., an evergreen perennial with white flowers, and *H. odorus* Waldst. & Kitt., a deciduous species with green flowers [21,22]. After pollination during fruit development, *H. niger* sepals also turn green and become photosynthetically active [23]. Both species develop flowers during late winter and early spring. Evergreen species have an advantage due to a longer vegetation period, while herbaceous species may use the advantage of green sepals to perform photosynthesis. Some authors claim that *H. odorus* leaves may survive winter. However, this is usually not the case in Slovenia [24]. We hypothesised that sepals of the two species will not only differ in chemical traits, but also in the photochemical efficiency of photosystem II (PS II) and in terms of their optical properties during the course of flower development.

## 2. Material and Methods

### 2.1. Sampling Locations

Plants were sampled at the foothills of Gobavica (latitude 46°9′57″ (46.1658); longitude 14°33′54″ (14.5651), 372 m a.s.l.) in the vicinity of Mengeš, Slovenia. To minimise the damage to the natural populations of the two species, plants were sampled at locations with high species abundance. Sampling took place three times during flower development, namely at the beginning of flowering (BBCH scale, stage 4), when flowers started to open, during the flowering period (BBCH scale, stage 6), and during fruiting (BBCH scale, stage 8). We randomly sampled 10 plants for each species and each developmental phase, which accounted for six different groups (three groups for three developmental phases × two groups for two studied species).

### 2.2. Sepal Morphological and Chemical Properties, and SLA

Specific sepal area (SLA) was determined as an area unit per dry matter (cm^2^ mg^−1^ DM). Discs with known diameter were cut from the sepals, dried in an oven (Memmert, Germany) at 105 °C for 24 h, and then weighed. Additional series of discs were used for chemical analyses.

Sepal stomata length and density were analysed on sepal impressions that were made using transparent nail polish, under 400× magnification and light microscopy (CX41; Olympus, Tokyo, Japan) with a digital camera (XC30; Olympus) and the CellSens Imaging Software 1.4.1 (Olympus).

The contents of chlorophyll *a*, chlorophyll *b*, and carotenoids in the sepals were determined according to Lichtenthaler and Buschmann [25,26]. The absorbance of acetone extracts (100% (*v*/*v*)) of the sepal samples was measured at 470 nm, 645 nm, and 662 nm with a UV/VIS spectrometer (Lambda 25; Perkin-Elmer, Norwalk, CT, USA). The content of anthocyanins in the sepals was determined following the procedure described by Drumm and Mohr [27]. The absorbance of acidified methanol extracts (methanol/HCl (37%) = 99:1 (*v*/*v*)) of the sepal samples was measured at 530 nm. Total methanol-soluble UV-B- and UV-A-absorbing substances were determined according to Caldwell [28]. Sepal samples were homogenised in methanol/distilled water/HCl (*v*/*v*/*v*) = 79:20:1 and centrifuged in a top-refrigerated centrifuge (1600×*g*, 10 °C, 10 min) after 20 min of incubation. The supernatants were decanted and scanned at intervals of 1 nm in the range from 280 nm to 319 nm for the UV-B-absorbing substances, and from 320 nm to 400 nm for the UV-A-absorbing substances. The extinction values were integrated separately for the UV-B and UV-A regions. All the chemical parameters are expressed per sepal area.

### 2.3. Sepal Optical Properties

Sepal optical properties were determined in the laboratory on vital sepals on the day of sampling. The reflectance and transmittance spectra were measured following the procedure described in Klančnik et al. [12]. Reflectance and transmittance were measured in the range from 300 nm to 800 nm, at a resolution of ~1.3 nm, using a portable spectrometer (Jaz Modular Optical Sensing Suite; Ocean Optics, Inc., Dunedin, FL, USA; grating, #2; slit size, 25 µm) with an optical fibre (QP600-1-SR-BX; Ocean Optics, Inc.) and an integrating sphere (ISP-30-6-R; Ocean Optics, Inc.). The sepal reflectance spectra were measured for the adaxial sepal surface by illumination with a UV/VIS-near infrared (NIR) light source (DH-2000; Ocean Optics, Inc.). The spectrometer was calibrated to 100% reflectance using a white reference panel with >99% diffuse reflectance (Spectralon; Labsphere, North Sutton, NH, USA). The reflectance spectra were calculated as the ratio of the sample data to the white reference. The sepal transmittance spectra were measured at the abaxial sepal surface by illumination of the adaxial sepal surface with the light source. The spectrometer was calibrated to 100% transmittance with a light beam that passed directly into the interior of the integrating sphere [29]. To obtain correlations between sepal traits and spectra, we divided the spectra into different bands (UV-B, UV-A, violet, blue, green, yellow, red, and near infrared (NIR)).

### 2.4. Statistical Analyses

Normal distributions of the data were evaluated using Shapiro–Wilk tests. Homogeneity of variance from the means was analysed using Levene’s test. The significance of the differences in sepal traits between the six studied groups for each measured parameter was assessed using one-way analysis of variance followed by Duncan’s post hoc multiple comparison tests. In addition, we performed Pearson’s correlation analysis to evaluate the relationships between the studied sepal traits. These analyses were performed using IBM SPSS statistics 22.0 (IBM, Armonk, NY, USA), with significance accepted at *p* ≤ 0.05. The figure for mean relative reflectance and transmittance spectra of the sepals was drawn in Microsoft Excel 2016 (Microsoft, Redmond, WA, USA). The significance of the differences in stomata density and size between the two *Helleborus* species was determined using Student’s *t*-tests in Microsoft Excel 2016 only for the fruiting phase. Significance was accepted at *p* ≤ 0.05. Redundancy analysis was used to explain the variance in sepal reflectance and transmittance spectra with sepal traits. The significance of the effects of the variables was determined using Monte Carlo tests with 999 permutations. Forward selection of the explanatory variables was used to avoid co-linearity between the variables. Non-significant variables (*p* > 0.05) were excluded from further redundancy analysis. All the variables used in the analysis were standardised.

## 3. Results

**Figure 1:**
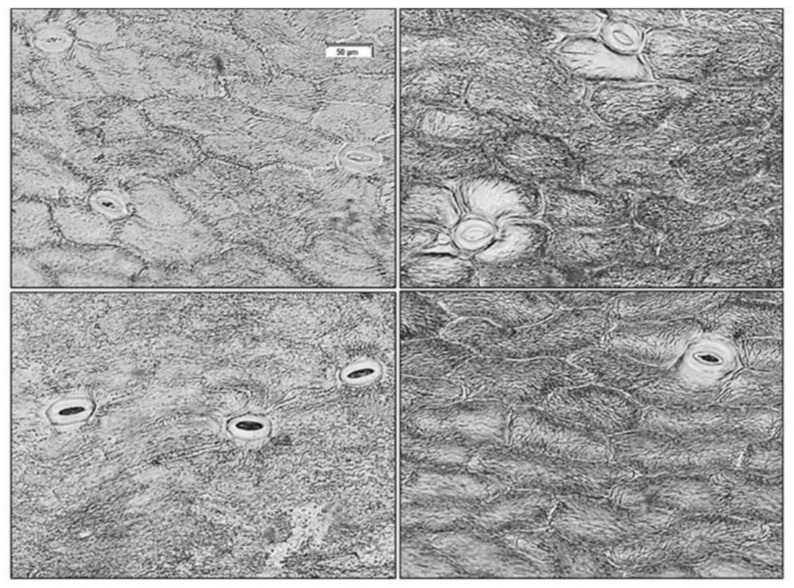
The imprints of sepal adaxial (**upper**) and abaxial (**lower**) surfaces of *Helleborus odorus* (**left**) and *H. niger* (**right**).

The imprints of sepal adaxial and abaxial surfaces of *H. odorus* and *H. niger* during the fruiting phase are shown in Figure 1. Stomata density (mm^2^) was significantly higher in *H. odorus* (16.7 ± 3.7 for the lower epidermis and 14.8 ± 3.6 for the upper epidermis) than in *H. niger* (11.4 ± 3.0 for the lower epidermis and 6.0 ± 1.8 for the upper epidermis). Stomata size (µm) was similar for the lower epidermis of both species (50.2 ± 4.5 in *H. odorus* and 50.1 ± 3.8 in *H. niger*), while it was significantly higher for the upper epidermis of *H. niger* (45.2 ± 5.3 for *H. odorus* and 51.9 ± 4.5 for *H. niger*).

The two species differed significantly in the contents of photosynthetic pigments and anthocyanins in the sepals, but less in their UV-absorbing substances’ contents (Table 1). Significant differences in photosynthetic pigment contents were also obtained within species between different developmental phases. The differences were more pronounced in *H. niger* in comparison to *H. odorus*. SLA decreased with sepal age. Additionally, sepal thickness increased with age. For *H. odorus*, the potential photochemical efficiency of PS II was high during all the three phases, while in the case of *H. niger*, it was initially low, but increased with sepal age (Table 1).

Differences in pigment contents affected sepal reflectance and the transmittance spectra. Reflectance was uniform in the UV region for both species in all phases (Table S1). However, the differences between the two species in the developing and flowering phases were significant in the visible regions. In the developing phase, spectral signatures became more alike. A similar pattern could be observed for the sepal transmittance spectra.

Overall correlation analysis revealed only few significant relationships. Thus, we performed correlation analyses separately for each developmental phase. Sepal reflectance was not significantly related to SLA, sepal thickness, and carotenoids in any of the three phases. We obtained some negative significant correlations between the visible and NIR regions and chlorophylls and anthocyanins, especially in the developing phase (Table 2). UV-B-absorbing substances seemed to be important in the flowering phase, while UV-A-absorbing substances were important in the developing and fruiting phases.

In the case of sepal transmittance in the developing phase, we obtained significant negative correlations between UV-A, violet, blue, yellow, and red with anthocyanins and chlorophyll *a* and *b*. UV-B-absorbing substances were more important in the flowering phase, while UV-A-absorbing substances and carotenoids were more important in the fruiting phase (Table 3). Both reflectance and transmittance were negatively related to the potential photochemical efficiency of PS II. However, these relations were only significant for the developing and fruiting phases (Table 2 and Table 3).

**Figure 2:**
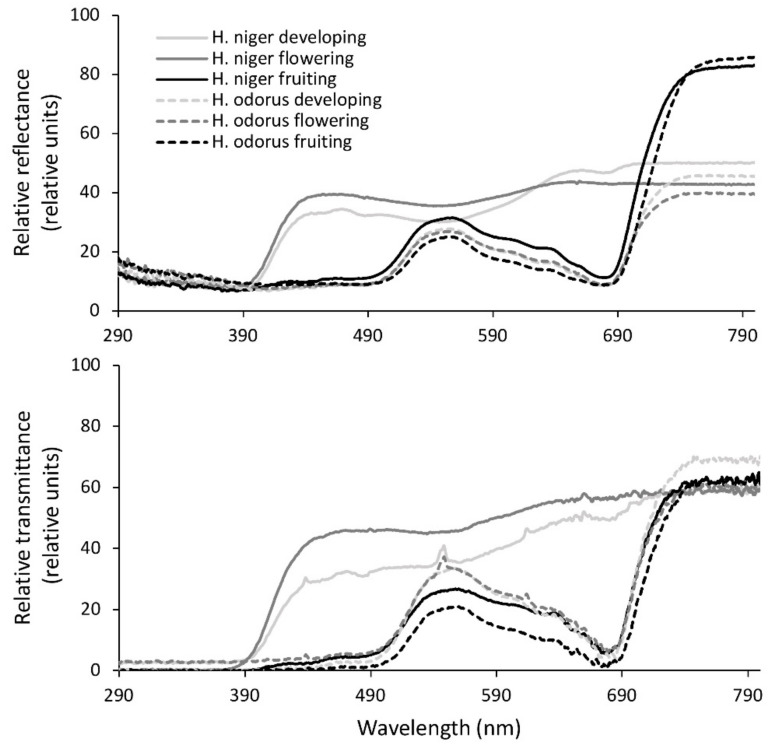
The curves for mean relative reflectance (upper graph) and transmittance spectra (lower graph) from 290 nm to 800 nm, as measured for *H. niger* and *H. odorus* sepals during the different developmental phases; data were smoothed using moving averages with a period of 5 consecutive measurements (*n* = 10).

The shapes of the reflectance curves of the green-coloured *H. odorus* sepals revealed typical green leaf spectra with peaks in the green and NIR regions, and low reflectance in the shorter (UV) wavelengths (Figure 2, upper graph). The reflectance of these sepals also showed an increase from UV-A towards UV-B. The initially white-coloured sepals of *H. niger* in the developing and flowering phases reflected an equal amount of light along the visible and NIR regions, while reflectance in the UV region was similar to that of *H. odorus*.

The transmittance spectra looked similar to the reflectance spectra. In the fruiting phase, the spectra resembled those typical of green leaves, as was the case for the *H. odorus* reflectance spectra. The differences between the two species could also be observed when comparing sepal transmittance spectra (Figure 2, lower graph). These were also similar for all the phases of *H. odorus* and for the fruiting phase of *H. niger*. The developing and flowering phases of *H. niger* sepals showed high transmittance above 350 nm, with a slightly increasing trend towards NIR. Transmittance in the UV region was close to zero.

**Figure 3:**
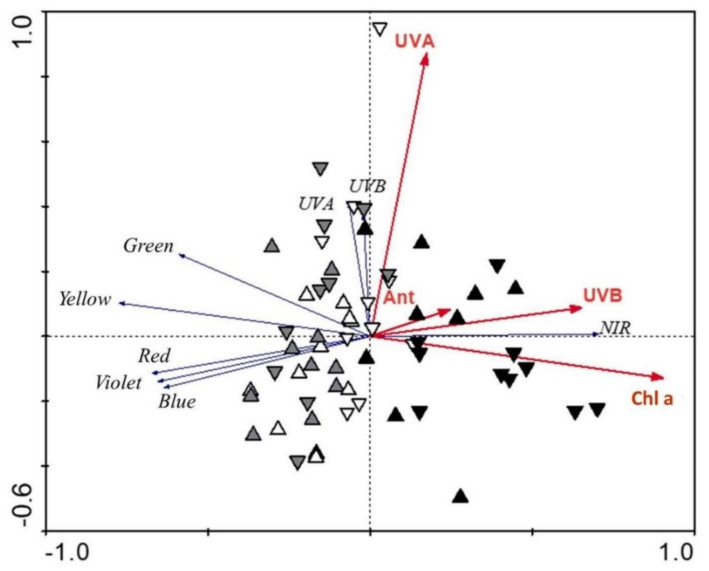
Redundancy analysis plot showing the strength of the associations between the sepal traits and different regions of the reflectance spectra for *H. niger* (up-triangles) and *H. odorus* (down-triangles). White triangles—developing phase; grey triangles—flowering phase; black triangles—fruiting phase. Only significant sepal parameters are shown. Chl a—chlorophyll *a*; Ant—anthocyanins; UVA—UV-A-absorbing substances; UVB—UV-B-absorbing substances.

An RDA plot showing the strength of the associations between the sepal chemical properties and the regions of the reflectance spectra (Figure 3) revealed the high importance of sepal chemistry in explaining leaf reflectance when examining simple effects. The potential to explain the reflectance spectra variability with single parameters (marginal effects) was 33% and 31% for chlorophyll *a* and *b*, while for carotenoids and UV-absorbing substances, it was 17% for each. When we analysed all parameters together (conditional effects), 32% of the reflectance spectra variability was explained with chlorophyll *a* (*p* = 0.001), while UV-A-absorbing substances, UV-B-absorbing substances, and anthocyanins explained an additional 4% (*p* ≤ 0.05) each. Altogether, we explained 44% of the reflectance spectra variability with the measured parameters. The vectors for chlorophyll *a* and anthocyanins are pointing in the opposite direction as vectors for the different regions of visible light. Symbols indicating different groups of sepals are distributed along different vectors. Symbols indicating sepals of the developing and flowering plants are mainly distributed along the UV-A-absorbing substances vector, while symbols indicating sepals of the fruiting *H. odorus* plants are distributed along the chlorophyll *a* vector. The vectors for chlorophyll *a* and UV-B-absorbing substances are related to the first axis, while the vector for UV-B-absorbing substances is related to the second axis.

**Figure 4:**
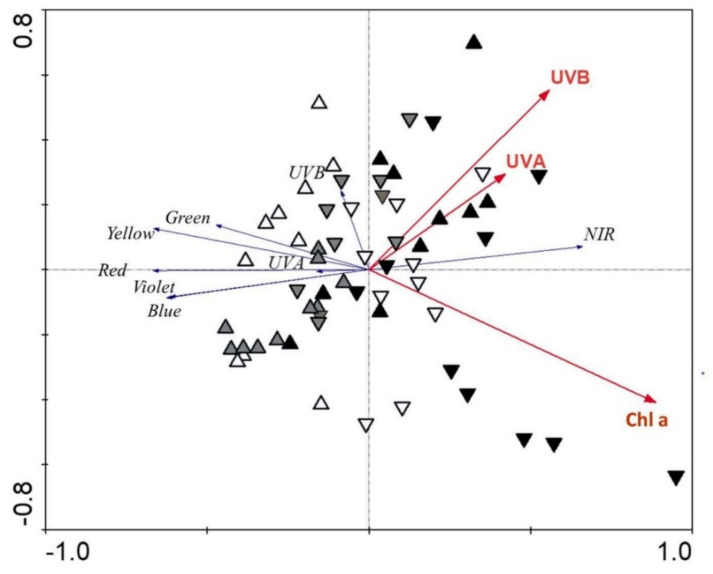
Redundancy analysis plot showing the strength of the associations between the sepal traits and different regions of the transmittance spectra for *H. niger* (up-triangles) and *H. odorus* (down-triangles). White triangles—developing phase; grey triangles—flowering phase; black triangles—fruiting phase. Only significant sepal parameters are shown. Chl a—chlorophyll *a*; UVA—UV-A-absorbing substances; UVB—UV-B-absorbing substances.

The potential to explain the transmittance spectra variability with single parameters (marginal effects) was 26% and 22% for chlorophyll *a* and *b*, respectively, while carotenoids explained 8%, and UV-B- and UV-A-absorbing substances explained 10% and 6%, respectively. When we tested all parameters together (conditional effects), we explained 26% of the transmittance spectra variability with chlorophyll *b* (*p* = 0.001), while UV-A-absorbing substances and UV-B-absorbing substances explained an additional 5% and 3%, respectively (*p* ≤ 0.05). Altogether, we explained 34% of the transmittance spectra variability with the measured parameters. The chlorophyll *a* vector opposes the vectors for the different regions of visible light (Figure 4). The vector for UV-A-absorbing substances opposes the vectors for UV-A and visible regions. Here, different groups of sepals are partly overlapping.

## 4. Discussion

Sepals of the two species showed differences in optical properties during their development. This was mainly due to the species-specific traits that determine their physical and chemical properties and, thus, the colour of the sepals. These traits also include photosynthetic pigments, which offer sepals the potential to perform photosynthesis. Salopek-Sondi et al. [30,31] claim that in *H. niger* and some other *Helleborus* species with coloured sepals, the selection during the evolution process is not related to floral function, but more likely to the transformation of these sepals into effective photosynthetic structures. This was also revealed by the presence of stomata in the sepals in our study, although their density in *H. odorus* and *H. niger* was relatively low in comparison to the leaves of mesophyte species [32]. In the case of *H. viridis* agg., the stomata density of sepals was 70–80% lower in comparison to leaves [33].

In *H. odorus* with green-coloured sepals, the potential for photosynthesis is permanently present. However, this is not the case in *H. niger*, which initially has white sepals. Therefore, its photosynthetic capacity increases during flower development, as also revealed from our measurements of the photochemical efficiency of PS II. In *H. viridis* agg. with green sepals, the electron transport rate was found to be 80% of that in mature leaves [33]. The sepals of *H. niger* become green or reddish only when the seeds of *H. niger* become ripen, since leucoplasts turn into chloroplasts after pollination [30]. The increase in chlorophyll *a* and *b* during flower development was significant not only for *H. niger*, but also for *H. odorus*. An increase in chlorophyll after fertilisation was also shown in the study of Brcko et al. [34], while in the ripe stage, the concentrations of chlorophyll gradually declined. This way, the photosynthetic capacity of sepals and other green parts of the flowers contributes to the process of seed ripening. This was also shown for *H. niger* [30] and *H. foetidus* [35]. During flower anthesis, the perianth of *H. foetidus* is tube-like, and it opens after pollination [16]. For *H. viridis*, contrasting results were obtained. Aschan et al. [33] showed that ripening fruits use respiratory carbon dioxide for photosynthesis and, thus, benefit in terms of the carbon budget. However, this was not the case in the study of Guitián and Larrinaga [16], who revealed that sepals persisting after flowering do not contribute to the development of seeds. High photosynthetic capacity of sepals in early phases, which we measured in *H. odorus*, is especially important, because this is an herbaceous species that has to use the advantage of favourable light conditions before the tree canopy closes [1].

Regarding sepal chemistry, the pattern of changes was different for the accessory pigments carotenoids. Sepal carotenoid contents gradually increased in both studied species and did not differ significantly among the two species in a certain phase. The same increasing trend was also obtained for anthocyanins and UV-B-absorbing substances, while the level of UV-A-absorbing substances was more or less the same during flower development. Similarly, a study of *H. niger* by Schmitzer et al. [20] also revealed an increase in total anthocyanin content in subsequent developmental stages. However, the contents of flavonols, which are absorbed in the UV region, were reduced during later developmental stages.

The chemical traits of sepals are also reflected in their optical properties. The reflectance and transmittance spectra in the two studied species had similar shapes, which confirmed the important role of sepal chemistry in shaping their optical properties, as shown by many other studies [12,36]. As expected, we obtained mainly negative relationships between the reflectance and transmittance spectra in the different regions and different sepal chemical traits, especially for anthocyanins and chlorophylls, as was also the case in other studies [5]. The strongest correlations were obtained in the early developmental phase. This is a consequence of pronounced differences in many sepal traits of the two studied species in this early phase. The greatest share of the reflectance and transmittance spectra was explained by chlorophyll *a*. The same importance could also be attributed to chlorophyll *b*, as shown by RDA as marginal effects. Other significant chemical parameters in explaining the variability of optical properties were UV-absorbing substances, especially UV-A-absorbing substances, the contents of which slightly increased during flower development.

Flower colouration is the primary pollinator attractor [37]. Flower colour is a result of its optical properties, namely light scattering and reflection by floral structures, and the absorption of selected wavelengths by pigments [38]. Most representatives of Hymenoptera that pollinate the representatives of the genus *Helleborus* have trichromatic colour vision, where maximal sensitivity values occur at 340 nm, 430 nm, and 535 nm, with an additional small peak at 600 nm [39]. The sepal spectral signatures of *H. niger* in the developing and flowering phases showed a slight increase at 340 nm and 650 nm, while in *H. odorus*, a clear peak in all three phases occurred around 540 nm, and some smaller peaks were evident at 600 nm and 660 nm. Flies that pollinate the representatives of the genus *Helleborus* also have colour vision. In addition, they have 3-hydroxyretinol as an antennal pigment, which broadens their spectral sensitivity even to the UV region [40]. This weak accordance between spectra peaks and insect vision thus confirms the suggestion of Salopek-Sondi et al. [30,31] that sepals of the *Helleborus* species are not evolving in the direction of floral function, but rather to serve as effective photosynthetic structures.

## 5. Conclusions

Sepals of *H. odorus* and *H. niger* went through chemical changes that led to changes in optical properties during their development. Sepal pigment contents, especially chlorophylls, explained 44% and 34% of the reflectance and transmittance spectra variability. Their photosynthetic capacity differed among the two species. In the white *H. niger*, it was low in the early phases of flower development, whereas it was permanently high in the green *H. odorus*, as revealed from photochemical efficiency of PS II. High photosynthetic capacity of sepals is especially important in the herbaceous species *H. odorus*, which uses the advantage of favourable light conditions before the tree canopy closes. However, this trait is less critical in the evergreen species *H. niger*. The high importance of chlorophyll *a* in explaining the reflectance and transmittance spectra, and the weak accordance between spectra peaks and insect vision support the claims of Salopek-Sondi et al. [30,31] that sepals of the *Helleborus* species are not evolving in the direction of floral function, but rather as photosynthetic structures.

## Figures and Tables

**Table 1:** *Helleborus odorus* and *H. niger* sepal traits for the different developmental phases.

Traits	Species and Developmental Phase					
	Developing Phase		Flowering Phase		Fruiting Phase	
	*H. odorus*	*H. niger*	*H. odorus*	*H. niger*	*H. odorus*	*H. niger*
Chlorophyll *a* (mg cm^−2^)	1.65 ± 0.36 ^b^	0.58 ± 0.29 ^a^	1.14 ± 0.50 ^ab^	1.53 ± 0.53 ^b^	4.51 ± 1.36 ^d^	3.14 ± 0.91 ^c^
Chlorophyll *b* (mg cm^−2^)	2.37 ± 0.84 ^b^	0.65 ± 0.68 ^a^	1.26 ± 0.39 ^a^	1.18 ± 0.44 ^a^	4.35 ± 2.00 ^c^	2.59 ± 0.78 ^b^
Carotenoids (mg cm^−2^)	0.37 ± 0.13 ^a^	0.34 ± 0.13 ^a^	0.66 ± 0.16 ^b^	0.71 ± 0.18 ^b^	1.01 ± 0.28 ^c^	1.08 ± 0.26 ^c^
Anthocyanins (au cm^−2^)	0.03 ± 0.01 ^b^	0.01 ± 0.01 ^a^	0.05 ± 0.02 ^c^	0.07 ± 0.02 ^cd^	0.06 ± 0.02 ^cd^	0.08 ± 0.02 ^d^
UV-B-AS (au cm^−2^)	0.58 ± 0.09 ^a^	0.58 ± 0.17 ^a^	0.65 ± 0.14 ^ab^	0.52 ± 0.09 ^a^	0.78 ± 0.20 ^bc^	0.88 ± 0.22 ^c^
UV-A-AS (au cm^−2^)	1.72 ± 0.14 ^b^	1.61 ± 0.07 ^a^	1.65 ± 0.10 ^ab^	1.59 ± 0.07 ^a^	1.65 ± 0.04 ^ab^	1.59 ± 0.11 ^a^
Sepal thickness (µm)	183± 34 ^a^	226 ± 61 ^bc^	194 ± 11 ^a^	205 ± 21 ^ab^	240 ± 17 ^cd^	266 ± 13 ^d^
SLA (cm^2^ mg^−1^)	1.05 ± 0.17 ^c^	1.18 ± 0.35 ^c^	1.04 ± 0.23 ^bc^	1.01 ± 0.23 ^abc^	0.84 ± 0.12 ^ab^	0.83 ± 0.09 ^a^
Fv/Fm (au)	0.77 ± 0.03 ^c^	0.31 ± 0.20 ^a^	0.76 ± 0.02 ^c^	0.45 ± 0.16 ^b^	0.79 ± 0.01 ^c^	0.79 ± 0.03 ^c^

Data are means ± SD; *n* = 10; different superscript letters within each row indicate significant differences between the means (*p* ≤ 0.05; Duncan’s tests); au, arbitrary units; SLA, specific sepal area; Fv/Fm, potential photochemical efficiency; UV-B-AS, UV-B-absorbing substances; UV-A-AS, UV-A-absorbing substances; NIR, near infrared.

**Table 2:** Correlations between sepal reflectance in the different regions of the spectrum and some of the other measured *H. niger* and *H. odorus* sepal traits for each of the three different developmental phases. Significant correlations are indicated in bold (*p* ≤ 0.05; Pearson’s correlations).

Sepal Traits		Fv/Fm	Anthocyanins	Chl. *a*	Chl. *b*	UV-B-AS	UV-A-AS
UV−B	D	0.58	0.35	**0.54**	**0.63**	0.14	0.17
	FL	**0.63**	−0.23	−0.25	0.11	**0.47**	0.35
	FR	−0.06	0.36	0.25	−0.28	0.29	**−0.56**
UV−A	D	0.43	0.17	0.34	0.43	0.27	0.23
	FL	**0.57**	−0.13	−0.17	0.05	0.43	0.37
	FR	−0.24	−0.36	−0.45	−0.31	−0.01	0.25
Violet	D	**−0.70**	**−0.61**	**−0.73**	**−0.57**	−0.06	**−0.50**
	FL	**−0.82**	0.37	0.33	−0.18	**−0.52**	−0.33
	FR	−0.26	**−0.48**	**−0.51**	−0.16	−0.09	0.44
Blue	D	**−0.71**	**−0.61**	**−0.72**	**−0.55**	−0.08	**−0.48**
	FL	**−0.84**	0.33	0.33	−0.19	**−0.51**	−0.35
	FR	−0.32	**−0.50**	**−0.54**	−0.22	−0.31	**0.48**
Green	D	−0.34	−0.38	−0.37	−0.16	−0.15	−0.34
	FL	**−0.73**	0.33	0.29	−0.30	**−0.45**	−0.35
	FR	−0.29	**−0.50**	**−0.52**	−0.08	−0.34	**0.55**
Yellow	D	**−0.64**	**−0.59**	**−0.66**	**−0.45**	−0.12	−0.43
	FL	**−0.80**	0.32	0.32	−0.24	**−0.48**	−0.41
	FR	−0.27	**−0.50**	**−0.50**	−0.03	−0.28	**0.58**
Red	D	**−0.85**	**−0.70**	**−0.85**	**−0.70**	−0.08	**−0.45**
	FL	**−0.85**	0.29	0.34	−0.15	**−0.50**	−0.41
	FR	0.10	0.26	0.23	0.22	−0.23	0.01
NIR	D	**−0.74**	**−0.62**	**−0.66**	−0.34	−0.04	−0.41
	FL	**−0.70**	0.02	0.13	−0.19	−0.34	−0.37
	FR	0.10	0.26	0.23	0.22	−0.23	0.01

Fv/Fm, potential photochemical efficiency; SLA, specific sepal area; AS, absorbing substances; NIR, near infrared; D, developing phase; FL, flowering phase; FR, fruiting phase.

**Table 3:** Correlations between sepal transmittance in the different regions of the spectrum and some of the other measured *H. niger* and *H. odorus* sepal traits for each of the three different developmental phases. Significant correlations are indicated in bold (*p* ≤ 0.05; Pearson’s correlations).

Sepal Traits		Fv/Fm	Anthocyanins	Chl. *a*	Chl. *b*	Carotenoids	UV-B-AS	UV-A-AS
UV-B	D	−0.30	0.10	−0.26	−0.25	0.23	**0.52**	−0.08
	FL	−0.39	0.28	0.41	0.10	0.37	−0.00	−0.36
	FR	−0.11	−0.02	−0.13	0.01	−0.26	−0.17	0.24
UV-A	D	**−0.58**	**−0.60**	**−0.65**	**−0.48**	−0.42	−0.32	−0.32
	FL	−0.07	0.21	0.16	0.03	−0.04	0.05	−0.18
	FR	−0.37	−0.35	−0.43	0.11	0.44	−0.33	**0.67**
Violet	D	**−0.75**	**−0.62**	**−0.78**	**−0.63**	−0.18	−0.04	−0.43
	FL	**−0.80**	0.36	0.30	−0.21	0.20	**−0.55**	−0.40
	FR	−0.34	−0.29	−0.38	0.20	**0.49**	−0.43	**0.73**
Blue	D	**−0.74**	**−0.65**	**−0.77**	**−0.61**	−0.20	−0.12	−0.42
	FL	**−0.83**	0.34	0.30	−0.22	0.21	**−0.56**	−0.39
	FR	−0.28	−0.32	−0.38	−0.06	0.40	−0.28	**0.55**
Green	D	−0.39	−0.31	**−0.46**	−0.23	−0.08	−0.07	−0.12
	FL	**−0.78**	0.37	0.26	−0.28	0.19	**−0.55**	−0.37
	FR	−0.25	−0.38	−0.43	0.02	0.42	−0.34	**0.63**
Yellow	D	**−0.61**	**−0.55**	**−0.67**	**−0.45**	−0.17	−0.17	−0.21
	FL	**−0.81**	0.39	0.30	−0.24	0.20	**−0.54**	−0.35
	FR	−0.26	−0.39	−0.44	0.11	**0.46**	−0.41	**0.71**
Red	D	**−0.77**	**−0.73**	**−0.80**	**−0.64**	−0.26	−0.26	−0.33
	FL	**−0.83**	0.37	0.34	−0.18	0.21	**−0.54**	−0.35
	FR	−0.09	−0.22	−0.16	−0.10	0.30	0.07	0.29
NIR	D	0.07	−0.03	0.06	0.12	**−0.49**	−0.27	0.43
	FL	−0.30	0.22	0.02	−0.10	0.44	−0.35	0.04
	FR	−0.03	0.34	0.26	−0.25	−0.29	0.29	**−0.57**

Fv/Fm, potential photochemical efficiency; SLA, specific sepal area; AS, absorbing substances; NIR, near infrared; D, developing phase; FL, flowering phase; FR, fruiting phase.

## Data Availability

Not applicable.

## Supplementary Materials

The following supporting information can be downloaded at: https://www.mdpi.com/article/10.3390/plants11010119/s1, Table S1: *Helleborus odorus* and *H. niger* sepal reflectance and transmittance in the different regions of the spectrum for the different developmental phases.
