# Supplementary material for: Comparative Study of Temporal Changes in Pigments and Optical Properties in Sepals of Helleborus odorus and H. niger from Prebloom to Seed Production
Source: Plants (Basel). 2021 Dec 31;11(1):119. doi: 10.3390/plants11010119 (PMC8747330; doi:10.3390/plants11010119)
Supplement: Supplementary file 1 [file plants-11-00119-s001.zip › plants-1527794-supplementary.pdf]

Table S1 *Helleborus odorus* and *H. niger* sepal reflectance and transmittance in the different regions of the spectrum for the different developmental phases.

	Species and developmental phase					
	Developing phase		Flowering phase		Fruiting phase	
**Traits**	*H. odorus*	*H. niger*	*H. odorus*	*H. niger*	*H. odorus*	*H. niger*
**Reflectance** (%)						
UV-B	13.08 ± 1.77^{b}	10.36 ± 1.14^{a}	14.39 ± 2.01^{c}	11.10 ± 0.81^{a}	14.83 ± 1.76^{c}	10.45 ± 0.73^{a}
UV-A	9.39 ± 1.29^{b}	8.23 ± 0.70^{a}	10.79 ± 1.31^{c}	9.16 ± 0.49^{b}	10.96 ± 1.28^{c}	7.85 ± 0.42^{a}
Violet	7.66 ± 1.00^{a}	25.57 ± 6.98^{b}	7.90 ± 1.02^{a}	30.28 ± 3.64^{c}	8.92 ± 1.04^{a}	9.43 ± 1.14^{a}
Blue	8.77 ± 1.19^{a}	33.18 ± 10.41^{b}	9.11 ± 1.81^{a}	38.76 ± 4.95^{c}	9.13 ± 0.86^{a}	11.09 ± 1.65^{a}
Green	22.53 ± 3.31^{ab}	31.10 ± 12.12^{cd}	22.08 ± 3.63^{ab}	36.25 ± 5.69^{d}	19.94 ± 1.87^{a}	26.10 ± 3.17^{bc}
Yellow	19.77 ± 3.55^{ab}	36.45 ± 10.11^{c}	20.09 ± 3.77^{ab}	39.28 ± 4.38^{c}	16.78 ± 1.51^{a}	24.24 ± 3.00^{b}
Red	13.18 ± 2.41^{a}	46.33 ± 5.39^{d}	13.74 ± 2.64^{a}	43.04 ± 3.42^{c}	11.80 ± 0.93^{a}	17.21 ± 2.35^{b}
NIR	43.84 ± 2.72^{ab}	49.93 ± 4.36^{b}	37.98 ± 2.09^{a}	42.78 ± 3.43^{ab}	78.43 ± 1.81^{c}	71.17 ± 22.4^{c}
**Transmittance** (%)						
UV-B	0.07 ± 0.07^{a}	2.65 ± 7.97^{a}	0.16 ± 0.05^{a}	0.26 ± 0.09^{a}	-0.02 ± 0.10^{a}	-0.04 ± 0.09^{a}
UV-A	0.06 ± 0.05^{a}	0.43 ± 0.28^{a}	0.10 ± 0.10^{a}	2.82 ± 4.92^{b}	-1.05 ± 3.15^{a}	0.10 ± 0.44^{a}
Violet	0.88 ± 0.52^{a}	22.81 ± 7.18^{b}	0.65 ± 0.35^{a}	33.62 ± 6.83^{c}	0.41 ± 0.32^{a}	2.36 ± 1.02^{a}
Blue	3.05 ± 1.75^{a}	32.73 ± 9.83^{b}	2.16 ± 0.94^{a}	45.98 ± 8.83^{c}	1.25 ± 0.73^{a}	4.81 ± 1.77^{a}
Green	25.42 ± 7.02^{b}	35.34 ± 13.15^{c}	22.14 ± 2.79^{b}	47.22 ± 8.28^{d}	15.20 ± 3.24^{a}	21.48 ± 3.83^{ab}
Yellow	23.60 ± 7.99^{b}	42.20 ± 12.44^{c}	21.54 ± 2.99^{b}	52.47 ± 9.14^{d}	13.43 ± 3.35^{a}	21.52 ± 3.44^{b}
Red	13.17 ± 5.70^{ab}	51.10 ± 12.90^{c}	11.73 ± 2.11^{ab}	56.92 ± 8.47^{c}	6.77 ± 2.17^{a}	13.87 ± 2.71^{b}
NIR	65.86 ± 6.69^{b}	62.45 ± 12.58^{ab}	56.74 ± 3.84^{a}	60.38 ± 6.01^{ab}	58.78 ± 3.42^{a}	59.60 ± 3.38^{ab}

Data are means ± SD; n = 10

Different superscript letters within each row indicate significant differences between the means ($p \leq 0.05$; Duncan's tests)

Reflectance and transmittance spectra represent means within 5-nm intervals ($p \leq 0.05$; Duncan's tests)

NIR, near infrared
